# Progress in the study of genome size evolution in Asteraceae: analysis of the last update

**DOI:** 10.1093/database/baz098

**Published:** 2019-10-14

**Authors:** Daniel Vitales, Pol Fernández, Teresa Garnatje, Sònia Garcia

**Affiliations:** 1 Institut Botànic de Barcelona (IBB, CSIC-ICUB), Passeig del migdia s/n, 08038 Barcelona, Catalonia, Spain; 2 Facultat de Biologia, Universitat de Barcelona, Avinguda Diagonal 643, 08038 Barcelona, Catalonia, Spain

## Abstract

The Genome Size in Asteraceae Database (GSAD, http://www.asteraceaegenomesize.com) has been recently updated, with data from papers published or in press until July 2018. This constitutes the third release of GSAD, currently containing 4350 data entries for 1496 species, which represent a growth of 22.52% in the number of species with available genome size data compared with the previous release, and a growth of 57.72% in terms of entries. Approximately 6% of Asteraceae species are covered in terms of known genome sizes. The number of source papers included in this release (198) means a 48.87% increase with respect to release 2.0. The significant data increase was exploited to study the genome size evolution in the family from a phylogenetic perspective. Our results suggest that the role of chromosome number in genome size diversity within Asteraceae is basically associated to polyploidy, while dysploidy would only cause minor variation in the DNA amount along the family. Among diploid taxa, we found that the evolution of genome size shows a strong phylogenetic signal. However, this trait does not seem to evolve evenly across the phylogeny, but there could be significant scale and clade-dependent patterns. Our analyses indicate that the phylogenetic signal is stronger at low taxonomic levels, with certain tribes standing out as hotspots of autocorrelation between genome size and phylogeny. Finally, we also observe meaningful associations among nuclear DNA content on Asteraceae species and other phenotypical and ecological traits (i.e. plant habit and invasion ability). Overall, this study emphasizes the need to continue generating and analysing genome size data in order to puzzle out the evolution of this parameter and its many biological correlates.

## Introduction

Genome size (GS) is a key biodiversity character, with significant evolutionary implications. While it is remarkably constant at the species level (1), there is a huge diversity in eukaryotes (2). Within plants, angiosperms are one of the groups showing the highest ranges of GS variation (ca. 2300-fold; 3). Since the early botanical studies on nuclear DNA amounts (e.g. 4, 5) there has been continuous and growing interest in the acquisition and analysis of GS data. The applications of measuring nuclear DNA amounts in evolutionary and ecological research are manifold. Perhaps the most extended use of GS in evolutionary studies is related to chromosome and ploidy variation, facilitating research on taxonomy, phylogeny and reproductive biology, among other fields (6). From the point of view of evolutionary ecology, many interesting correlates have been found at different levels (see for example 7), among which life cycle and invasiveness are two of the most intensively studied (e.g. 8, 9). Beyond the relevance of GS as a biological character, its knowledge has also a practical application: it is an essential information for planning genome sequencing projects (10) or others involving techniques such as amplified fragment length polymorphisms (AFLP) fingerprints (11) or microsatellites (12). Finally, one of the factors that have contributed the most to the increase of nuclear DNA amounts information in the recent years is the availability, relative ease of use and price drop of flow cytometry (FC) (13). Due to the continuous and increasing interest in GS data, several electronic databases have been developed, covering major groups of organisms including animals, fungi and plants (e.g. 14, 15, 16). In plants, the reference source of GS is the plant DNA C-values database curated by researchers at Kew Gardens (17) running from September 2001.

Following on from our own research work on GS in family Asteraceae, we developed the GSAD ‘Genome Size in Asteraceae Database’ in 2010 (18), which is the only GS database centred in a particular plant family. The sunflower family is one of the most intensely investigated taxonomic groups. Research involving directly or indirectly GS in Asteraceae are plentiful. Recently, several research projects have studied Asteraceae using phylogenomic approaches (19, 20), for which data on nuclear DNA amounts are essential. In the same way, GS data have been key information for recent studies focusing on repeatome evolution in various groups of species within this family (e.g. 21, 22, 23). Last but not least, a few Asteraceae genomes have already been sequenced and assembled (i.e. horseweed, *Erigeron canadense*, 24; sunflower, *Helianthus annuus*, 25; artichoke, *Cynara scolymus*, 26; lettuce, *Lactuca sativa,*^27^; sweet wormwood, *Artemisia annua*, 28), but many more are planned for the near future (20) and nuclear DNA amount will be basic prior knowledge for this purpose. The study intensity in Asteraceae is explained because of its large size, since it is most likely the largest of angiosperms (ca. 24 700 species), and worldwide distribution (29). Besides, many Asteraceae have economic interest as foods (i.e. sunflower, artichoke, lettuce), medicinal (i.e. chamomile, sweet wormwood), ornamentals (i.e. daisy, marigold, etc.) or invasive species (i.e. common ragweed, diffuse knapweed, narrow-leaved ragwort, etc.) and this has triggered their deeper study than in other plant families, despite no model plants belong to Asteraceae.

Global scientific production is under exponential growth, with estimations of the number of publications doubling every 24 years (30), and research involving GS in family Asteraceae has not been an exception. In this scenario, databases obtaining information from published sources need to regularly update to continue being useful. Since its release, the GSAD ‘Genome Size in Asteraceae Database’ was only updated by July 2013 (3). The present work focuses on the last update, which contains information published until July 2018 and represents a 57.72% data increase in only 5 years. However, despite much data have been accumulated, the study of GS variation at family level has been largely ignored from a phylogenetic standpoint. Therefore, taking advantage of the important data rise on the last release of the GSAD, we performed a comprehensive study involving phylogenetically independent tests and evolutionary signal analyses to explore the diversity and evolution of GS in Asteraceae. Finally, we also studied some classic correlations between GS and some phenotypical and ecological traits (i.e. plant habit and invasion ability) and we identify knowledge gaps in the family to promote further research.

## Materials and Methods

### Third GSAD update: data collection

We have used web search engines (Scopus, ISI Web of Knowledge and Google Scholar) with combinations of the keywords ‘genome size’ or ‘nuclear DNA amount’ or ‘nuclear DNA content’ and (‘Asteraceae’ or ‘Compositae’) and looking for the keywords in abstract, title and keywords. This strategy has proved useful (31), reducing the amount of documentary noise and increasing the specificity. Only articles published in periodic journals or book chapters were considered as information sources. Genome size data extracted from the articles were compiled in an Excel file (Microsoft, Redmond, WA, USA), including complete bibliographic reference to the original source. Genome size information has been complemented with other data (i.e. chromosome number, ploidy level, life cycle, tribe and subfamily of each species), coming either from the source publication, from other databases (such as the Chromosome Counts Database, http://ccdb.tau.ac.il/home/) or from specific floras or Asteraceae treatments (29). To delimitate invasive species in the database we used the Global Invasive Species Database (http://www.iucngisd.org/gisd/), searched for all the Asteraceae invasive species and looked for coincident species in our database.

Finally, we have applied the concept of h-index to the ‘genome size & Asteraceae (or Compositae)’ topic. The h index (32) is a popular bibliometric indicator which combines productivity (number of documents) and impact (number of citations) in one index and, although it is mostly aimed to appraise individual researchers, its approach can also be used to evaluate the current interest of a certain research topic. To compare h-indices between Fabaceae, Brassicaceae, Poaceae, Orchidaceae and Asteraceae we used Scopus options (https://www.scopus.com/) with the same combination of terms described, changing the family names for the corresponding ones: (‘Fabaceae’ or ‘Leguminoseae’ or ‘Papilionaceae’), (‘Brassicaceae’ or ‘Cruciferae’), (‘Poaceae’ or ‘Gramineae’) and (‘Orchidaceae’). The searches were performed through the platform Scopus (https://www.scopus.com/) and h-indices calculated by ordering the articles resulting from each query by descending number of citations.

### Statistical and phylogenetic analyses

We have conducted statistical analyses both including and excluding phylogenetic relationships among taxa. Because there were much less DNA sequences available than species in the database, we selected a representative set of taxa for the tree construction with which to perform phylogenetic analyses. However, since that meant a significant reduction in the number of taxa that could be analysed (from 806 to 134 regarding diploid individuals) we also present basic statistical analyses (i.e. without considering the phylogenetic relationship among the species) considering the whole dataset.

All data manipulations and statistical analyses were performed with RStudio IDE, v.0.98.1078 (http://www.rstudio.com/), a user interface for R (33). Analyses of regression and Shapiro–Wilk test for normality were performed. One-way analysis of variance (ANOVA) test and *t*-test were calculated when possible, while in those cases where datasets were not normally distributed, we performed non-parametric tests such as Spearman rank correlation, the Kruskal–Wallis test by ranks and multiple comparison tests after Kruskal–Wallis (using the ‘pgirmess’ package for R). In addition, to analyse relationships among GS (2C), chromosome number and life cycle in a phylogenetic context, the phylogenetic generalized least squares (PGLS) algorithm, as implemented in the ‘nlme’ package for R, was used as in (34). The data from chromosome number and life cycle normally come from the GS source publication, and when absent, from the Chromosome Counts Database and/or available floras.

In order to perform the statistical–phylogenetic analyses, a phylogenetic tree was constructed with sequences of *mat*K (835 bp), *trn*L*-trn*F (1148 bp) and *rbc*L (702 bp) chloroplastic regions (total: 2685 bp), downloaded from GenBank and listed in Supplementary Data. One species per genus was used in most cases (although sometimes sequences from different species had to be used to represent a genus) and the analysis was conducted using modal and mean values for chromosome numbers (2*n*) and GS, respectively. The resulting tree included 134 genera belonging to 20 tribes and 6 subfamilies. *Nastanthus* (Calyceraceae) and *Menyanthes* (Menyanthaceae) were chosen as outgroup following (35). All taxa included in these analyses were diploid. The three sequence matrices obtained with the three molecular markers were edited with MAFFT, corrected manually and concatenated with Mesquite v.3.02 (36). The phylogenetic analyses were performed in the CIPRES Science Gateway (37). Bayesian inference phylogenetic analysis was performed in MrBayes v.3.2.6 (38) using the GTR + I + G model previously determined from jModeltest v.2.1.6 (39) under the Akaike information criterion (AIC; 40). Four consecutive MCMC computations were run for 100 000 000 generations, with tree sampling every 10 000 generations. The first 25% of tree samples were discarded as the burn-in period. Posterior probabilities (PP) were estimated through the construction of a 50% majority rule consensus tree.

We assessed the evolution of GS values through ancestral state reconstruction methods implemented in the R package ‘phytools’ (41), using the majority rule consensus tree obtained from Bayesian inference analysis. We calculated the mean value of GS per genus and conducted maximum likelihood (ML) ancestral state inference with four models of continuous trait evolution: white-noise (WN, absence of phylogenetic signal), Brownian model (BM, random drift), Ornstein–Uhlenbeck model (OU, a selective-adaptive model) and Early Burst (acceleration-deceleration of BM variance). Models were compared by using the corrected AIC as implemented in the R package ‘geiger’ (42). We used then the function ‘fastAnc’ in ‘phytools’ to infer ancestral character states by maximum likelihood at each node in the phylogeny and the function ‘contMap’ to plot these continuous character traits onto the phylogeny in ‘phytools’. The same procedures were employed to assess the evolution of the DNA amount per chromosome (2C/2*n*).

**Table 1:** Summary of the data present in the GSAD ‘A genome size in Asteraceae Database (Release 3.0)’

**Subfamily** **and tribe**	Mean^*^ (pg)	**Max (pg)**	**Min (pg)**	**Mean** **2C/2*n***	**Number of species**	**Number of species in GSAD**	**% representation**
**Asteroideae**	**8.54**	**65.50**	**0.47**	**0.308**	**15 500**	**851**	**5.49%**
Anthemideae	11.08	65.50	2.17	0.411	1800	355	19.72%
Astereae	3.75	21.43	0.47	0.250	3080	80	2.60%
Bahieae	4.84	4.84	4.84	0.242	85	1	1.18%
Calenduleae	3.27	5.69	1.75	0.108	270	7	2.59%
Coreopsideae	7.76	56.56	1.43	0.141	550	45	8.18%
Eupatorieae	3.33	7.20	0.79	0.166	2200	15	0.68%
Gnaphalieae	4.30	17.60	1.11	0.131	1240	57	4.60%
Helenieae	6.87	10.22	4.10	0.208	124	3	2.42%
Heliantheae	9.74	43.48	2.08	0.355	1500	86	5.73%
Inuleae	2.34	7.34	1.12	0.113	687	43	6.26%
Madieae	2.97	3.13	2.80	0.233	ca. 200	2	1.00%
Millerieae	5.24	11.50	0.98	0.144	400	31	7.75%
Perytileae	2.66	2.66	2.66	0.074	81	1	1.23%
Polymnieae	5.40	5.40	5.40	0.180	3	1	33.33%
Senecioneae	7.39	52.30	0.79	0.210	3500	123	3.51%
Tageteae	2.40	2.40	2.40	0.050	270	1	0.37%
**Barnadesioideae**	**8.50**	**8.55**	**8.44**	**0.469**	**91**	**2**	**2.20%**
Barnadesieae	8.50	8.55	8.44	0.469	91	2	2.20%
**Carduoideae**	**3.53**	**28.94**	**0.73**	**0.147**	**ca. 2600**	**267**	**10.27%**
Cardueae	3.53	28.94	0.73	0.147	2360	267	11.31%
**Cichorioideae**	**5.29**	**65.50**	**0.80**	**0.398**	**ca. 2900**	**363**	**12.52%**
Cichorieae	5.25	65.50	0.80	0.440	ca. 1500	317	21.13%
Vernonieae	6.41	39.90	1.58	0.123	ca. 1100	46	4.18%
**Gochnatioideae**	**3.40**	**4.53**	**2.27**	**0.052**	**88**	**1**	**1.14%**
Gochnatieae	3.40	4.53	2.27	0.052	88	1	1.14%
**Mutisioideae**	**6.04**	**7.90**	**2.19**	**0.104**	**630**	**11**	**1.75%**
Mutisieae	6.10	7.90	2.19	0.125	200	8	4.00%
Nassauvieae	5.89	7.80	3.66	0.093	300	3	1.00%
**Pertyoideae**	**1.82**	**1.82**	**1.82**	**0.07**	**50**	**1**	**2.00%**
Pertyeae	1.82	1.82	1.82	0.07	50	1	2.00%
**Asteraceae**	6.50	65.50	0.47		ca. 24 700	1496	6.06%

^*^means for each subfamily calculated considering the whole dataset.

To track the phylogenetic signal, the R package ‘phylosignal’ (43) was used to contrast the phylogenetic tree with the GS and chromosome number data (pruning the genera of which we did not have either information). The local Moran’s index (I*_i_*) was used to test whether closely related taxa tend to display similar GS values as a consequence of their phylogenetic proximity (43). This calculation, based on the concept of autocorrelation, allows us to discriminate whether a group in the phylogenetic tree exhibits strong conservation for certain high or low values of GS. The same method was employed to calculate local Moran’s index for the DNA amount per chromosome (2C/2*n*). In order to locate the evolutionary signal along the taxonomic levels we calculated the phylogenetic correlogram for GS using the function ‘phyloCorrelogram’ implemented in ‘phylosignal’ package. The package ‘ape’ was also required for the phylogenetic-statistical analyses. Finally, the phylogenetic signal was also evaluated using evolutionary approaches [i.e. Pagel’s λ (44) and Blomberg’s K (45)] estimated with ‘phylosig*’* function on ‘phytools’.

## Results

**Figure 1:**
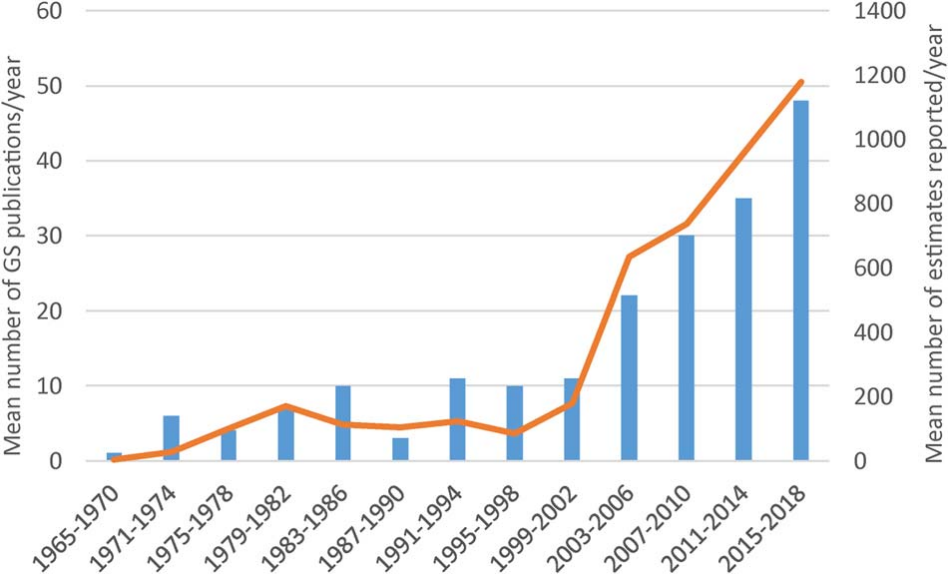
Mean number of Asteraceae genome size estimates reported per year over 13 successive 4-year periods between 1965 and 2018, the first period comprising 6 years. Data taken from GSAD ‘Genome Size in Asteraceae Database’ (Release 3.0, July 2018).

The third release of the GSAD database compiles information from 4350 accessions from 1496 plant species and 231 genera. A detailed summary of these data is presented in Table 1. The update represents an increase in source articles of 48.87% (from 133 articles consulted until release 2.0 to 198 articles in release 3.0), new data entries constituting a 36.60% of total GS estimations (Figure 1). The taxonomic coverage increased in 275 new species (22.52%) and 45 genera (24.19%) that were measured for the first time. The database includes information for 7 subfamilies and 24 tribes, representing an addition of one subfamily (Pertyoideae) and four tribes (Bahieae, Helenieae, Perytileae and Pertyeae). The best represented subfamily in terms of species is Cichorioideae (12.52%) and the tribe with more coverage among them is Cichorieae (21.13%). Regarding the other two major subfamilies, the Asteroideae and Carduoideae, the most represented tribes are the Anthemideae (19.72%) and the Cardueae (11.31%), respectively, taking into account only tribes with more than 10 species. The most represented genus is *Taraxacum* which has increased in a 606.67% since Release 2.0, basically attributable to a study that generated 637 entries on GS data for *Taraxacum officinale* (46). Other genera like *Hieracium*, *Crepis*, *Senecio* and *Helianthus* follow in representation, retaining the same order at the top of the list—although after *Taraxacum*—from Release 2.0 (Table S1). With regards to the technique used for GS estimation, the predominance of FC over other methodologies is clear: it is used in 86% of the total entries and 96.6% of the data in the last update were obtained with this technique.

With the Scopus search options, we found 146 articles on ‘genome size’ and ‘Asteraceae’ (or ‘Compositae’) that have been cited 3067 times since 1974 in total, representing an h-index of 32 for this topic. Compared to GS studies in other large angiosperm families, we found lower h-indices in all of them (i.e. Fabaceae, *h* = 25; Brassicaceae, *h* = 25; Orchidaceae, *h* = 20), except in Poaceae (*h* = 41).

### Diversity and distribution of C values in Asteraceae

The summary values of GS in each subfamily and tribe within Asteraceae are listed in Table 1. Excluding the measure of *Chrysanthemum lacustre,* which could be considered unreliable (47), holoploid nuclear DNA amount values in the family varied 139-fold, ranging from 2C = 0.47 pg (*Erigeron canadense*) to 65.5 pg (*Crepis barbigera*). At subfamily level, the Carduoideae is the subfamily with the lowest mean GS (2C = 3.53 pg) while Asteroideae have the highest mean GS value (2C = 8.54 pg), if we consider only groups with data for at least 10 species. Kruskal–Wallis test showed significant GS differences among subfamilies, both considering the whole dataset (*K* = 199.2, df = 6, *P* < 2.2e-16) or only diploid species (*K* = 96.36, df = 4, *P* < 2.2e-16). Multiple comparison test revealed significant GS differences between the larger subfamilies (i.e. Asteroideae, Cichorioideae and Carduoideae), while non-significant differences were found considering less represented subfamilies (i.e. Mutisioideae, Gochnatioideae, Barnadesioideae and Pertyoideae). At the tribe level, the Anthemideae (subfamily Asteroideae) has the highest mean GS value (2C = 11.08 pg), with the values ranging 65-fold. The tribe Inuleae, also within Asteroideae, shows the lowest mean GS (2C = 2.34 pg). Within this large subfamily the Kruskal–Wallis test showed GS differences among the tribes with data for at least 10 species (*K* = 305.22, df = 9, *P* < 2.2e-16). The multiple comparison test revealed that the Anthemideae, Heliantheae and Senecioneae show significantly higher 2C values than most of the other tribes, while Astereae, Inuleae, Gnaphalieae, Eupatorieae, Calenduleae and Millerieae show lower GS values (not shown).

**Figure 2:**
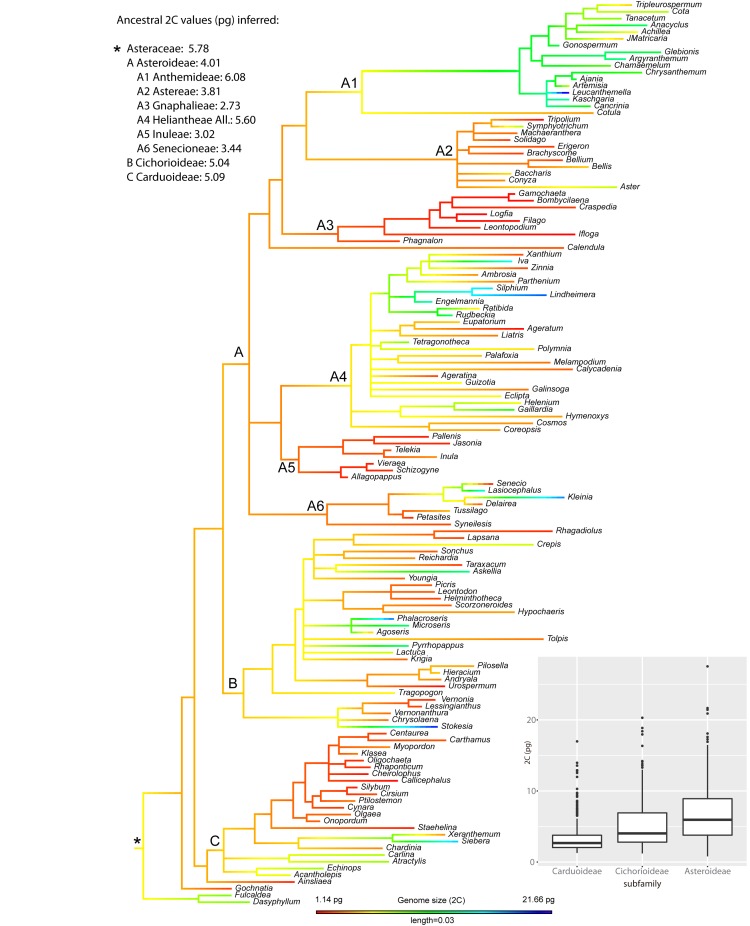
Ancestral genome size (2C) reconstruction in Asteraceae, indicating the ancestral value for the whole family (*) as well as for the best represented subfamilies (i.e. Carduoideae, Cichorioideae and Asteroideae) and tribes within the Asteroideae. Box plot shows the distribution of 2C values across the largest subfamilies, with horizontal lines representing median values and whiskers standard deviation.

As for the phylogenetic reconstruction, the resulting tree topology was overall consistent with currently accepted Compositae supertree phylogeny (35). All included subfamilies showed strong support (PPs between 0.96 and 1; Figure S1). The most important tribes were also reconstructed as monophyletic and highly supported. Regarding the best-fit model for GS evolution in the family, all tested models were supported—but the greatest strength of evidence was for the simplest Brownian motion (Table S2). Figure 2 shows the nuclear DNA contents (2C) mapped onto the phylogenetic tree of Asteraceae, inferring ancestral state reconstruction and representing ancestral 2C values for the main subfamilies and tribes. The most recent common ancestor (MRCA) of Asteraceae was reconstructed with a 2C = 5.78 pg under a maximum likelihood (ML) approach. At subfamily level, relatively similar ancestral values were reconstructed for the MRCA of Carduoideae (2C = 5.09 pg), Cichorioideae (2C = 5.04 pg) and Asteroideae (2C = 4.01 pg). At the tribe level, the MRCA of the Anthemideae was inferred as having 2C values of 6.08 pg, in contrast to the smallest ancestral 2C values reconstructed for Gnaphalieae (2C = 2.73 pg) or Inuleae (2C = 3.02 pg), all of them within subfamily Asteroideae.

Our analyses of phylogenetic signal indicate that related Asteraceae taxa have a significant tendency to resemble each other in terms of GS. Both autocorrelation (i.e. C mean and Moran’s I) and evolutionary approaches (i.e. Pagel’s λ and Bloomberg’s K) employed to estimate the phylogenetic signal showed significant relationship among GS values and phylogenetic history (Table S3; *P* < 0.05 in all cases). Figure S2 shows local Moran’s index (I*_i_*) values for GS calculated for each genus plotted onto the phylogeny. Significant I*_i_* values are present in most members of tribe Cardueae (61%), all the genera in tribe Gnaphalieae, most of Inuleae (75%), and most of Anthemideae (72%) in our tree. Therefore, this analysis of local phylogenetic signal (Figure S2) reveals hotspots of autocorrelation in four clades: the tribes Inuleae, Gnaphalieae and Cardueae (with low values of GS) and the subtribe Anthemideae (showing high values of GS). Phylogenetic correlogram analysis detected significant positive autocorrelation of GS values occurring at distances shorter than 0.027 substitutions per site (Figure S3). When highlighting this phylogenetic distance value over the heatmap of pairwise patristic distances among taxa, we observe that the strongest autocorrelation signal mainly corresponds to the taxonomic levels of tribes and below (Figure S4).

**Table 2:** Summary of the test results for the prediction of positive association between GS (2C, pg) and chromosome number (2*n*) across major clades of Asteraceae in both a phylogeny-dependent (Spearman rank correlation) and phylogeny-independent (PGLS) context

	**N. taxa**	***rho***	***P*-value**	
**Asteraceae**				
All taxa	1266	0.2057	>0.0001	^***^
Diploid taxa only	763	−0.1891	0.9999	
Phylogenetic dataset	128	-	0.4403	
**Carduoideae**				
All taxa	250	0.1909	0.0024	^**^
Diploid taxa only	195	0.0885	0.2182	
Phylogenetic dataset	22	-	0.7086	
**Cichorioideae**				
All taxa	334	0.1626	0.0028	^**^
Diploid taxa only	203	−0.2359	0.9996	
Phylogenetic dataset	30	-	0.2510	
**Asteroideae**				
All taxa	678	0.2840	>0.0001	^***^
Diploid taxa only	364	−0.0516	0.8371	
Phylogenetic dataset	73	-	0.6574	

Spearman rank correlations were applied to all taxa and to diploid taxa datasets; PGLS test were only applied to diploid taxa for which sequence information was available (i.e. phylogenetic dataset).

**Table 3:** Summary of the statistical analysis performed to test the association among genome size and life cycle (annuals or perennials) on taxa included in GSAD

		**Mean GS (2C)**				
	**N. taxa**	**Annuals**	**Perennials**	***K***	***P*-value**	
**Asteraceae**						
All taxa	1106	6.69	7.90	5.11	0.02374	^*^
Diploid taxa only	576	5.38	6.05	10.04	0.00153	^**^
Phylogenetic test	94	4.71	5.44	-	0.6599	
Phylogenetic dataset	94	4.71	5.44	1.87	0.1724	
**Carduoideae**						
All taxa	198	3.71	4.13	0.01	0.9541	
Diploid taxa only	144	3.58	4.03	0.34	0.5572	
**Cichorioideae**						
All taxa	266	7.20	5.41	11.65	0.00064	^***^
Diploid taxa only	130	4.95	5.95	1.51	0.2187	
**Asteroideae**						
All taxa	531	6.67	9.60	38.51	5.447e-10	^***^
Diploid taxa only	301	6.04	7.27	16.28	0.0000545	^***^

At family level, we tested the association considering all taxa and diploid taxa only (Kruskal–Wallis test) and considering the phylogenetic relationships (PGLS) on diploid taxa for which sequence information was available. For comparative purposes, the phylogenetic dataset was also analysed without taking into account the phylogeny (Kruskal–Wallis test). The phylogenetic tests have not been performed at the subfamily level.

**Figure 3:**
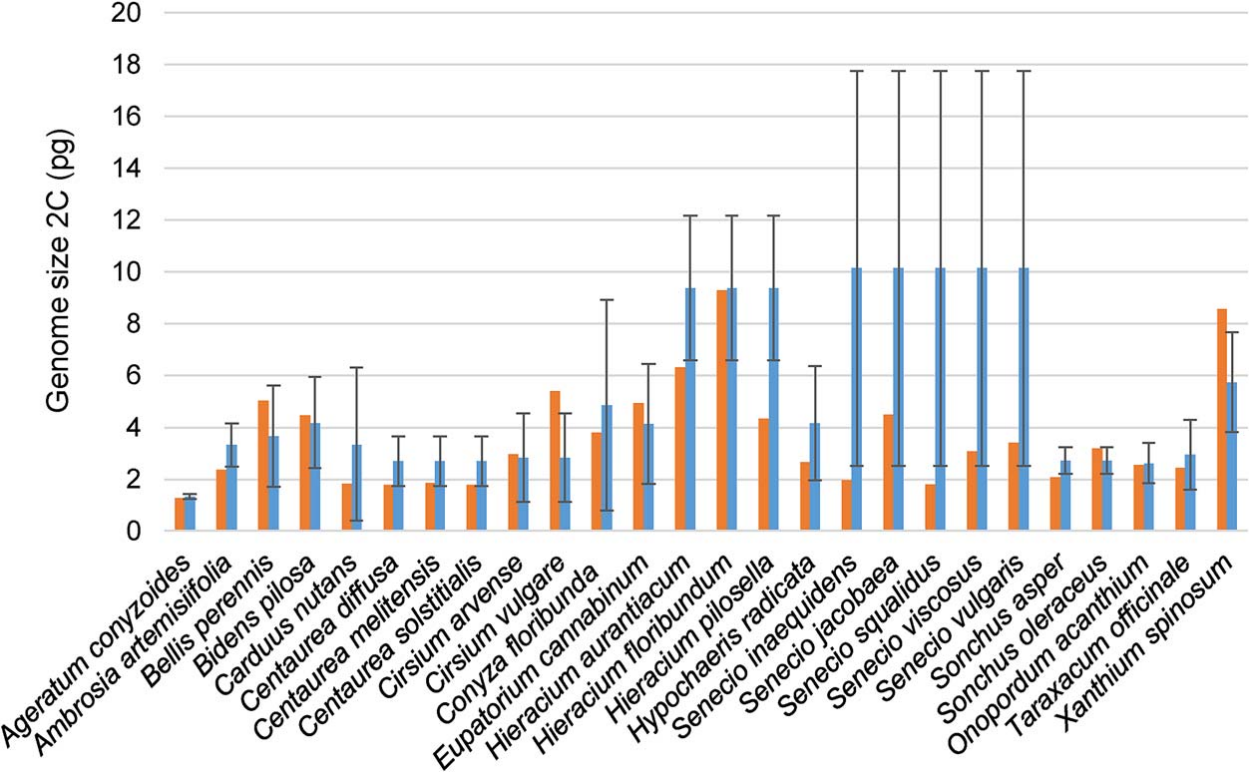
Genome size of the invasive species included in GSAD and the mean GS values of their respective genera (red and blue bars, respectively). Error bars represent SD obtained from the GS values of the genera.

### Correlations with chromosome number and ploidy level

The ploidy level ranges from 1*x* to 22*x* and chromosome number from 4 to 198 being the most common number 2*n* = 18 (19%). From the information available in the database, 701 (46.86%) of the species are only diploid, 293 (19.58%) are considered only polyploid and 74 (4.95%) are both diploid and polyploid, while the remaining 428 (28.61%) would be cases of unknown ploidy. A significant positive correlation between chromosome number and 2C values (*P* < 0.0001; *rho* = 0.2057) was found analysing the whole family. However, considering only diploids, there was no significant association between chromosome number and 2C-value, even if we take into account the phylogeny (Table 2). Similarly, positive correlation (*P* < 0.05 in all cases) between 2*n* and 2C was found within all the major subfamilies (i.e. Carduoideae, Asteroideae, Cichorioideae; see Table 2). Again, considering only diploid taxa, no significant association was detected among the GS and the number of chromosomes within any of the subfamilies, even taking into account the phylogeny (Table 2).

Significant differences for the DNA amount per chromosome (2C/2*n*) were found between subfamilies, both analysing the whole dataset (*K* = 198.52, df = 4, *P* < 2.2e-16) or the diploid taxa (*K* = 183.34, df = 3, *P* < 2.2e-16). Multiple comparison test revealed significant 2C/2*n* differences between Carduoideae (0.147 pg/chromosome) and the other large subfamilies (i.e. Asteroideae, 0.308 pg/chromosome; Cichorioideae, 0.398 pg/chromosome), while non-significant differences were found on the other cases. Among Asteroideae tribes with data for at least 10 species, the Kruskal–Wallis test also showed significant GS differences (*K* = 283.64, df = 9, *P* < 2.2e-16). The multiple comparison test revealed that Anthemideae and Heliantheae are the only tribes showing significantly higher 2C/2*n* values, while Inuleae, Gnaphalieae and Eupatorieae present significantly lower values (not shown). Phylogenetic signal calculations revealed that the DNA amount per chromosome shows a significant relationship with phylogeny (*P* > 0.05 for C mean, Moran’s I, Pagel’s λ and Bloomberg’s K; see Table S3). Here again, the best-fitting model of evolution selected according to AIC_C_ was the Brownian motion (Table S2). Figure S5 shows the DNA amount per chromosome (2C/2*n*) mapped onto the phylogenetic tree of Asteraceae, inferring ancestral state reconstruction and representing ancestral values for the main subfamilies and tribes. The MRCA of Asteraceae was reconstructed with a 2C/2*n* = 0.277 pg/chromosome under an ML approach. At subfamily level, relatively similar ancestral values were reconstructed for the MRCA of Carduoideae (2C/2*n* = 0.258 pg/chromosome), Cichorioideae (2C/2*n* = 0.315 pg/chromosome) and Asteroideae (2C/2*n* = 0.214 pg/chromosome). Within the large Asteroideae subfamily, the MRCA of the tribe Anthemideae was inferred as having 2C/2n values of 0.343 pg/chromosome, in contrast to the smallest ancestral 2C/2*n* values reconstructed for Gnaphalieae (2C/2*n* = 0.150 pg/chromosome) or Inuleae (2C/2*n* = 0.155 pg/chromosome). Figure S6 shows local Moran’s index (I*_i_*) values for the DNA amount per chromosome (2C/2*n*) calculated for each genus plotted onto the phylogeny.

### Genome size, life cycle and invasiveness

Considering the whole dataset, GS in annual plants is significantly different than in perennials (average 2C = 6.69 vs. 7.91 pg, respectively; *P* = 0.02138). Taking into account only diploid accessions, we also found significant differences in the GS among annual and perennial plants (average 2C = 5.38 vs. 6.06 pg, respectively; *P* = 0.00153). The same trend (i.e. significantly smaller GS values in annual than in perennial taxa) was observed when phylogenetic relationships were taken into account, but the association resulted non-significant (*P* > 0.05). However, note that when phylogenetic relationships are considered the dataset is reduced by ca. 90% (see Materials and Methods). At subfamily level, Asteroideae showed as well significantly smaller GS values in annuals than in perennials, both considering all accessions or only diploid taxa (see Table 3). The subfamily Carduoideae followed the same trend but lacking significant differences. In contrast, Cichorioideae showed the opposite trend [i.e. larger GS values in annuals (2C = 7.21 pg) than in perennials (2C = 5.41 pg)] considering all accessions, while no significant relationship was detected analysing only diploid taxa (Table 3).

Out of the 50 invasive species of Asteraceae currently recognized in the Global Invasive Species Database (consulted in February 2019), the GSAD contains GS information for 30 of them. These species, belonging to seven different tribes from the three major subfamilies (i.e. Carduoideae, Cichorioideae and Asteroideae), showed a mean GS of 2C = 3.57 pg (i.e. considerably lower than the mean value of the family, 2C = 6.50 pg). For those cases where many species of the genus had been measured, we also tested the differences between the GS of these invasive species included in GSAD and the mean GS values of their respective genera (Figure 3). Our results indicate significantly lower GS (Wilcoxon rank-sum test; *P* = 0.01019) in invasive taxa (average 2C = 3.61 pg) than the mean values of their respective genera (average 2C = 5.32 pg).

## Discussion

### Interest in genome size data in Asteraceae is steadily growing

The increase in entries makes this last update comparable to the first release of the database in amount of data added (1775 and 1592 new entries, respectively, in the first and third release). Moreover, we appreciate the same trend in the increase of the number of publications (Figure 1) that means that this topic is becoming more popular, possibly due to its new applications (e.g. in NGS projects) and numerous correlations with phenotypic or ecological traits, among others. The application of the h-index to the ‘genome size & Asteraceae’ topic corroborates our analysis of the literature. The high h-index for Asteraceae is remarkable given the absence of model plants in this family as compared to Fabaceae, Brassicaceae and Poaceae, whose presence undoubtedly contributes to their respective values. With respect to the journals that more frequently publish Asteraceae GS research, these are ‘Plant Systematics and Evolution’ (15), ‘Caryologia’ (6) and ‘Plant Biology’ (6), but there are 76 additional journals releasing papers on this topic. This means that there is a huge dispersion of the data publication and underlines the need of the GSAD database.

In the GSAD there are tribes such as Anthemideae, Chicorieae and Cardueae that are considerably better represented than most of the others (Table 1). Potential explanation for this bias is species richness (47). However, there are other Asteraceae tribes with comparable (or even higher) number of species but showing much lower representation in GSAD. Other reasons for this bias could be related to the geographic distribution of these tribes. For instance, Anthemideae, Cichorieae and Cardueae are largely abundant in Europe (35), where most of the research on plant GS has been taken place to date. The need of fresh material to perform the flow cytometric assessment (i.e. by far the most extended methodology; see below) could enhance this geographic bias. Finally, intensity of study can also explain this bias, i.e. the fact that certain research groups particularly active in certain tribes (maybe with certain economic interest) contribute a lot of GS data to these particular groups.

The percentage of polyploid taxa in the GSAD is also clearly biased in relation to their representation in the family. While many Asteraceae species are considered to be polyploid, their preponderance in the GSAD is striking: 51.2% of the species with ploidy level information belong to polyploid or presumed polyploid taxa. The presence of polyploid taxa is high in tribes Anthemideae, Cichoriae and Cardueae so this could partly explain this bias too. In relation to the first release of the database, the representation of polyploids has also doubled (from 25.5% in GSAD 1.0 to 51.2% in GSAD 3.0) highlighting the continued interest of researchers studying whole genome duplication processes. Finally, regarding the measurement techniques, we observed a clear tendency to favouring FC over other methodologies which are usually more tedious (e.g. Feulgen densitometry, biochemical methods). In the previous releases of GSAD the estimates derived from FC constituted 75.39% of the total entries while in the new measurements included in the last release 97.86% of the data are determined by FC. Recently, genome size estimations based on NGS projects are becoming more popular (21).

### Genome size, ploidy level and chromosome number

The significance in terms of data volume contributed by this third release of GSAD allowed more thorough analysis of GS diversity in Asteraceae than previously. These include ancestral reconstruction and phylogenetic signal analyses, to better understand the evolution of this trait along the family. Our results highlight the large variability of GS values in Asteraceae, with 2C-values ranging 139- fold, making it considerably more diverse in terms of GS than other large Eudicot families (e.g. Brassicaceae; 76-fold variation; Fabaceae, 33-fold variation; Rosaceae, 36.5-fold variation) according to (17). According to our data, a significant part of the differences in GS within the family is related to changes in chromosome number (Table 2). However, considering only diploid species, while the variability of GS values in Asteraceae is also large (ranging 41.11-fold), no significant correlation between chromosome number and 2C values was detected. These results suggest that the role of chromosome number in GS diversity within Asteraceae is basically related to polyploidy, while dysploidy would only cause minor variation in the DNA amount along the family. Similar patterns had already been reported in Asteraceae (47, 48) as well as in other groups of plants (e.g. 49).

Among diploid taxa of Asteraceae, the evolution of GS shows a strong phylogenetic signal, which best adjusts to a Brownian motion model. This result suggests that neutral selection (i.e. genetic drift) probably governed most of GS evolution on the family level. The reconstruction of ancestral GS values along the phylogenetic history of Asteraceae illustrates the evolution of this trait (Figure 2). The ancestor of the family may have had a medium-sized genome, with relatively poor variation at low taxonomic levels. A progressive increase of GS diversification likely occurred at higher taxonomic levels, coinciding with the divergence of major subfamilies and tribes. The significant GS differences observed among Carduoideae, Cichorioideae and Asteroideae subfamilies—as well as between tribes within the large Asteroideae subfamily—co-occur during the diversification events of those groups. The inferred dynamics of GS evolution mirror the results obtained for the ancestral reconstruction of 2C/2*n* values (Figure S2), suggesting that—at least among diploid taxa—GS divergence is likely driven by changes in DNA amount per chromosome. In *Lilium*, (50) also reported Brownian model of evolution for GS together with a significant correlation between GS and average chromosome length, consistent with the hypothesis that repetitive DNA may be the primary contributor to the GS diversity. In fireflies (51) a significant correlation was found among transposable element (TE) abundance and GS, this last trait also showing a neutral Brownian model of evolution. Indeed, in the absence of recent polyploidy, differential proliferation of TEs has been proposed as the major contributor to GS variability (52, 53). In Asteraceae, the only study focusing on the repeatome evolution at family level (21) inferred a positive correlation between GS and TE abundance. However, the sampling on that study was certainly limited (i.e. 15 species from 10 genera along the family), preventing a detailed description of TE dynamics and their relationship with GS evolution.

The above-mentioned phylogenetic signal analyses clearly point out the presence of a general association between GS and phylogeny in family Asteraceae. However, these approaches make the assumptions that traits evolve similarly across the phylogeny, while there are solid evidences that phylogenetic signal is scale dependent and varies among clades (43). The phylogenetic correlogram of genome size in Asteraceae exhibited a positive autocorrelation for short lags (Figure S3), indicating that the phylogenetic signal of this trait is significantly stronger at low taxonomic (i.e. within-tribe) levels (Figure S4). Local patterns on GS evolution were easy to characterize within the large and well-represented Asteroideae subfamily. Within this group, we found tribes showing significantly lower GS values (i.e. Gnaphalieae or Inuleae) together with the tribe showing the largest GS values on the whole family (i.e. Anthemideae) in which there was a particularly strong autocorrelation signal. Our ancestral trait reconstruction detected large differences for the MRCA among these groups (Figure 2), suggesting that GS values were evolutionary defined from the early diversification of those tribes. This result was confirmed by the local autocorrelation analyses (Figure S2) indicating significant local association in GS values for most of the members within these tribes, i.e. GS of the species are partly explained by their phylogenetic position within these tribes. Interestingly, very similar results were obtained from ancestral reconstruction and local autocorrelation analyses based on 2C/2*n* data (Figures S5 and S6). This suggest that the generally small GS in Gnaphalieae or Inuleae as well as the large GS in Anthemideae are likely related to evolutionary dynamics of DNA amount per chromosome (or impacting more or less evenly each chromosome). Specific repeatome changes at the early divergence of these clades could explain such strong phylogenetic signal on those groups. The observed variation in GS between tribes could be mainly driven by changes in the abundance of one single repeat family e.g. in Fabales (54) or by the global dynamics of several components of the repeatome e.g. in *Fritillaria* (10). Further genomic study of Asteroideae, including extensive repeatome characterization, will help elucidate the details explaining such contrasting GS evolution within this subfamily.

### Life cycle and invasiveness: are there any correlates with GS?

Both taking into account the whole dataset (i.e. including diploids and polyploids) and analysing only diploid species, we found a significant trend in which annual plants show smaller GS than taxa with perennial life cycle. This pattern had already been stated in Asteraceae (see 47 and references therein) and these results are confirmed with our 57.72% enlarged dataset. However, considering the phylogeny in the analyses, we found that the relationship among GS and life cycle was not significant (Table 3). These results might suggest that the observed association between GS and life cycle could be explained by the phylogenetic relationships among taxa. This phylogenetic bias linked to certain life cycle could explain the absence of significant association in Carduoideae, or even the negative correlation between GS and life cycle in Cichorioideae. However, the analysis of the same species included in the phylogenetic dataset but without considering the evolutionary relationships also resulted in a non-significant association among GS and life cycle. Therefore, we cannot discard that employing larger sampling in the phylogenetic analyses could result in a significant relationship between GS and life cycle, as we observed for the tests based on the whole dataset.

Regarding invasiveness, our results indicate that Asteraceae weeds show generally low GS values, tending to present significantly smaller GS than their congeners. Cells with faster divisions tend to have significantly less GS (e.g. 55) and plants with r strategy have less GS (e.g. 56). Nevertheless, it should be noticed that we found six invasive species (*Bellis perennis*, *Bidens pilosa*, *Cirsium arvense*, *Cirsium vulgare, Sonchus oleraceus* and *Xanthium spinosum*) showing larger DNA amounts than the mean values of their respective genera. Some of these species are polyploids (*Bidens pilosa* = 4*x* – 6*x*, *Cirsium vulgare* = 4*x* and *Sonchus oleraceus* = 4*x*), which could be also related to their invasive abilities. Bennett (57) proved that in closely related species polyploid individuals have a faster rate of meiosis and minimum generational time is also shorter. The study of the other 20 Asteraceae invasive species reported in GSAD (2019) but currently lacking GS information would be definitive to confirm the association among GS and invasiveness in the family.

### Conclusions and future perspectives

Although the study of genome size evolution in Asteraceae already has a considerable history, the interest of scientists on this topic has continued increasing in the recent years. Indeed, our analyses based on the latest update of the GSAD database have provided us novel insights regarding the evolutionary patterns of genome size in this family, as well as meaningful associations with ecological traits such as life cycle or invasiveness. These findings highlight the importance of continuously generating new GS measures, together with their collection in databases and the meta-analyses that can be carried out on them. Finally, our work points out the need to perform further comprehensive studies on repeatome and karyological diversity at the family level to better understand the evolution of genome size in Asteraceae.

## Author Contributions

SG, TG, and DV designed the study. SG and PF collected the data. SG, PF, and DV performed the analyses and drafted the manuscript. All authors contributed to the manuscript review.

## Supplementary Material

Fig_S1_Database_baz098

Fig_S2_Database_baz098

Fig_S3_Database_baz098

Fig_S4_Database_baz098

Fig_S5_Database_baz098

Fig_S6_Database_baz098

Vitales_et_al_Supplementary_Tables_Database_baz098
