# Supplementary figures and images for: Progress in the study of genome size evolution in Asteraceae: analysis of the last update
Source: Database (Oxford). 2019 Oct 14;2019:baz098. doi: 10.1093/database/baz098 (PMC6790504; doi:10.1093/database/baz098)

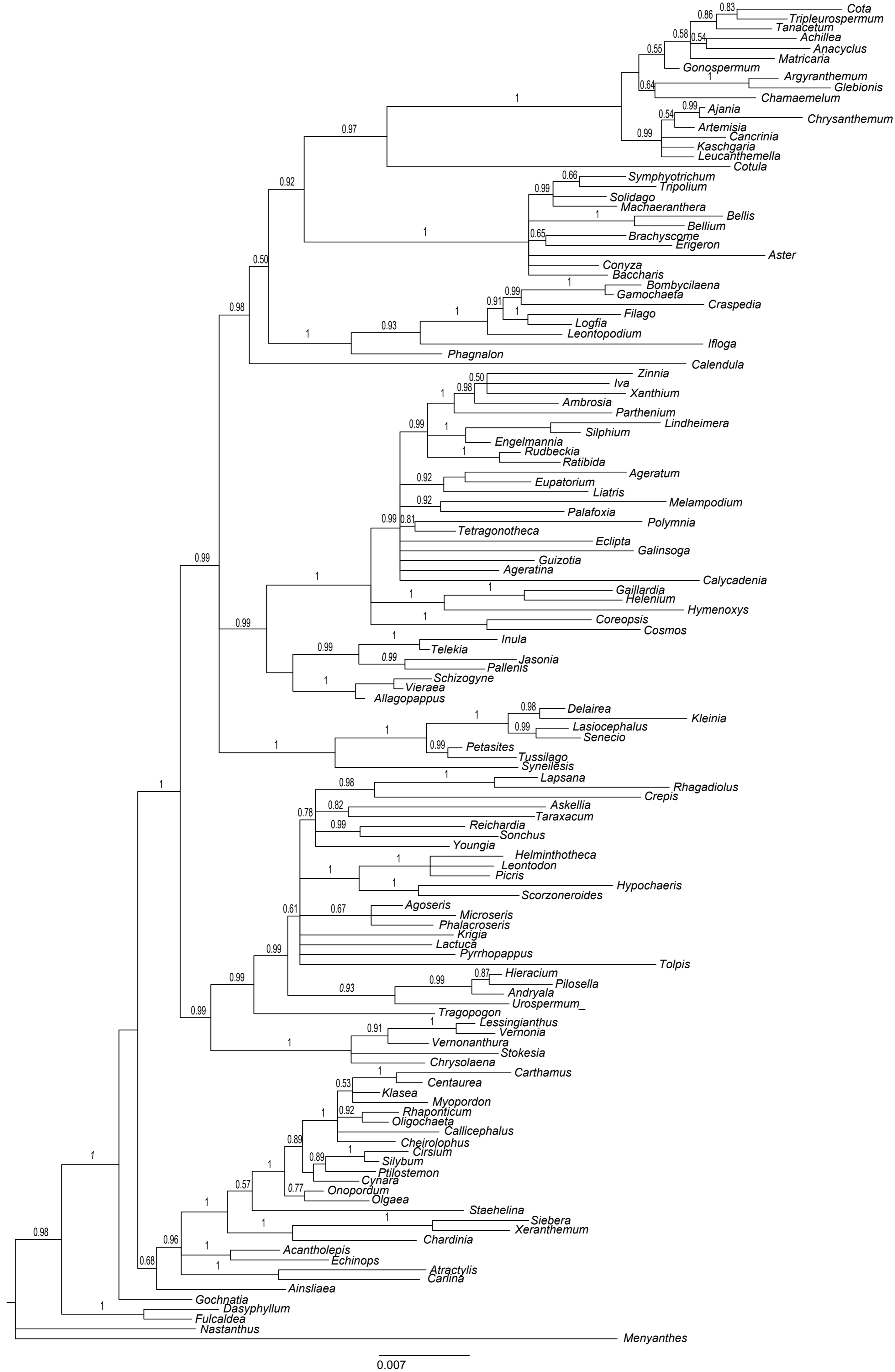

Supplement: Fig_S1_Database_baz098 [file fig_s1_database_baz098.pdf]

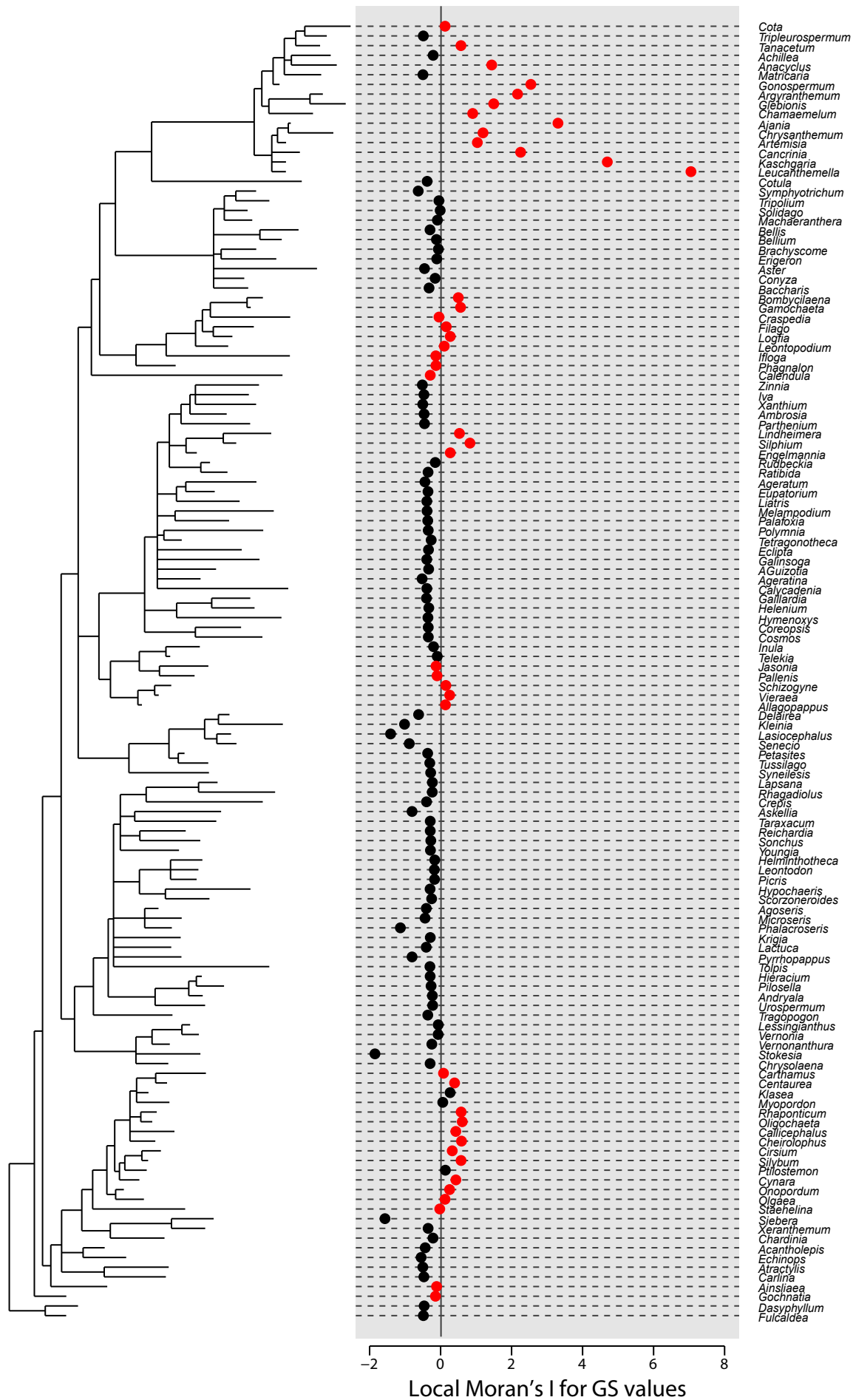

Supplement: Fig_S2_Database_baz098 [file fig_s2_database_baz098.pdf]

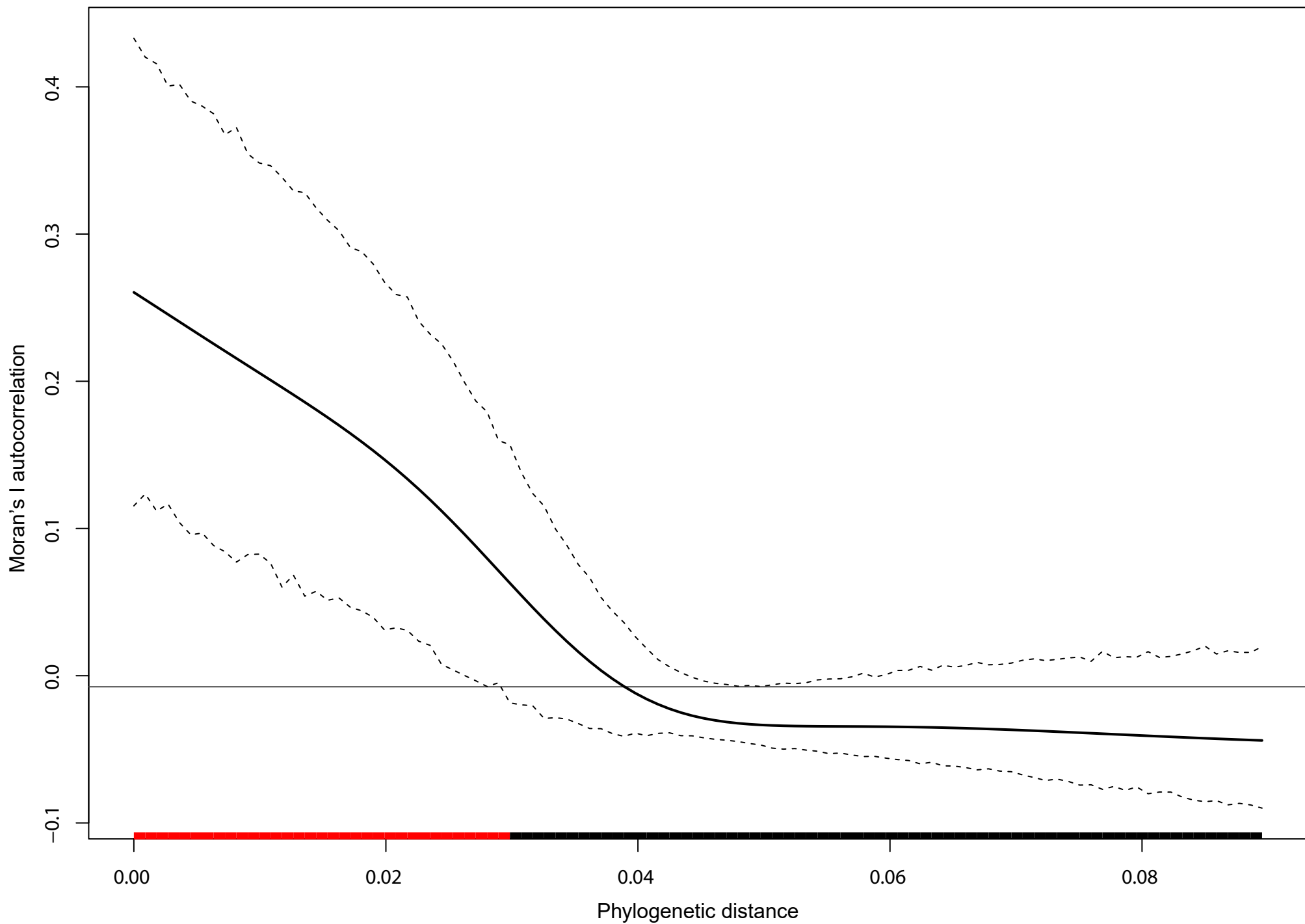

Supplement: Fig_S3_Database_baz098 [file fig_s3_database_baz098.pdf]

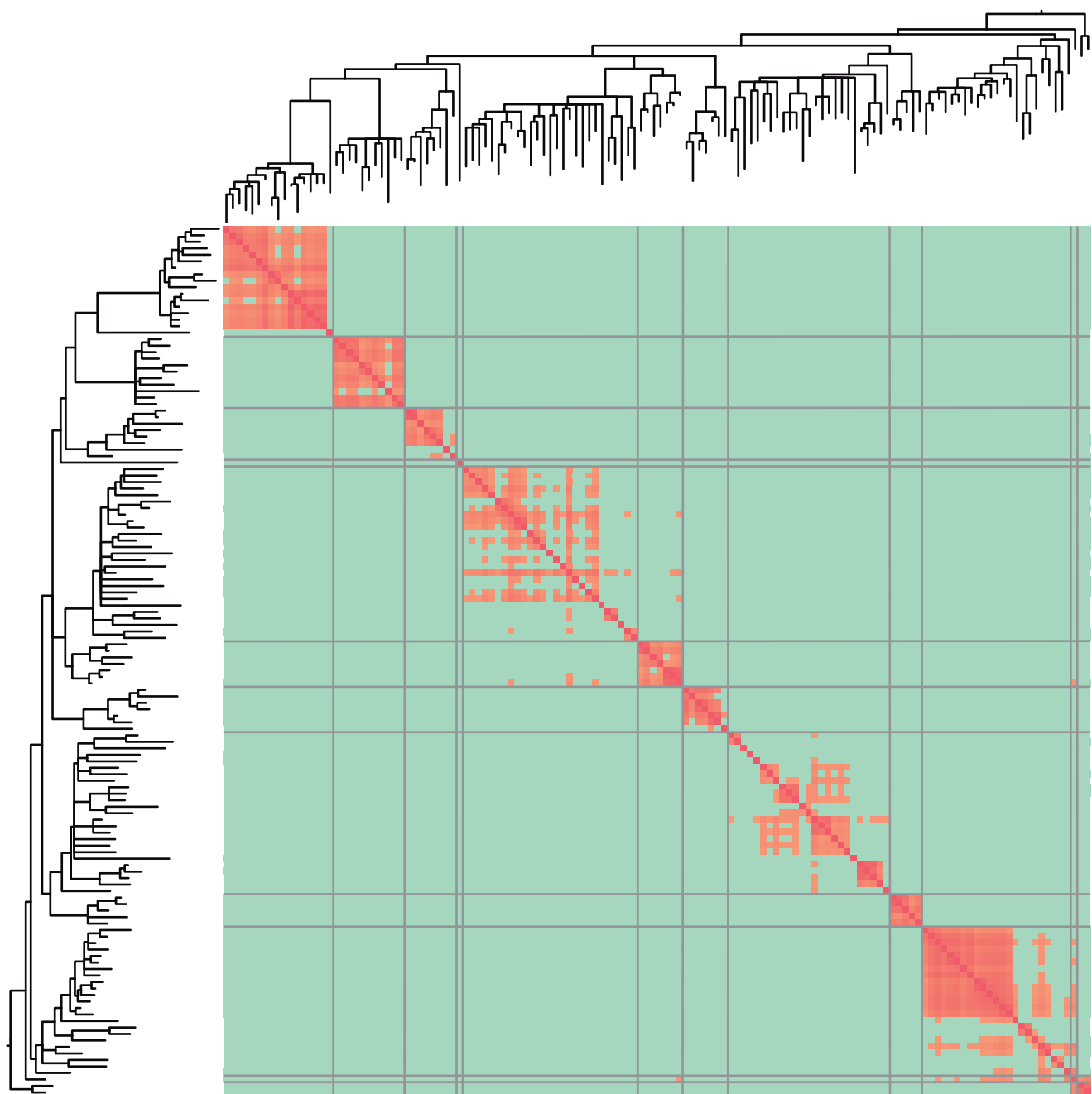

Supplement: Fig_S4_Database_baz098 [file fig_s4_database_baz098.pdf]

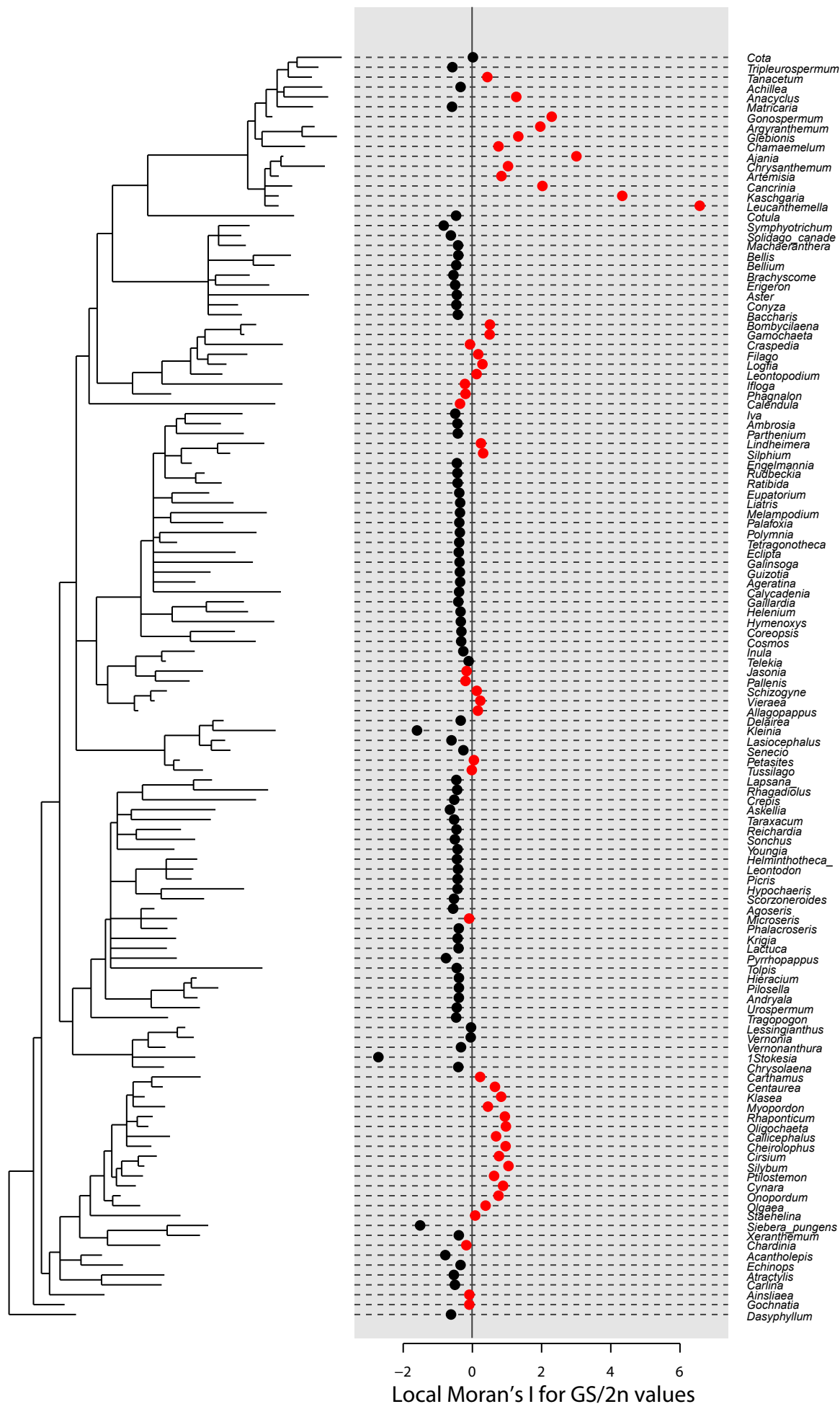

Supplement: Fig_S6_Database_baz098 [file fig_s6_database_baz098.pdf]
