# Supplementary material for: Progress in the study of genome size evolution in Asteraceae: analysis of the last update
Source: Database (Oxford). 2019 Oct 14;2019:baz098. doi: 10.1093/database/baz098 (PMC6790504; doi:10.1093/database/baz098)
Supplement: Fig_S5_Database_baz098 [file fig_s5_database_baz098.pdf]

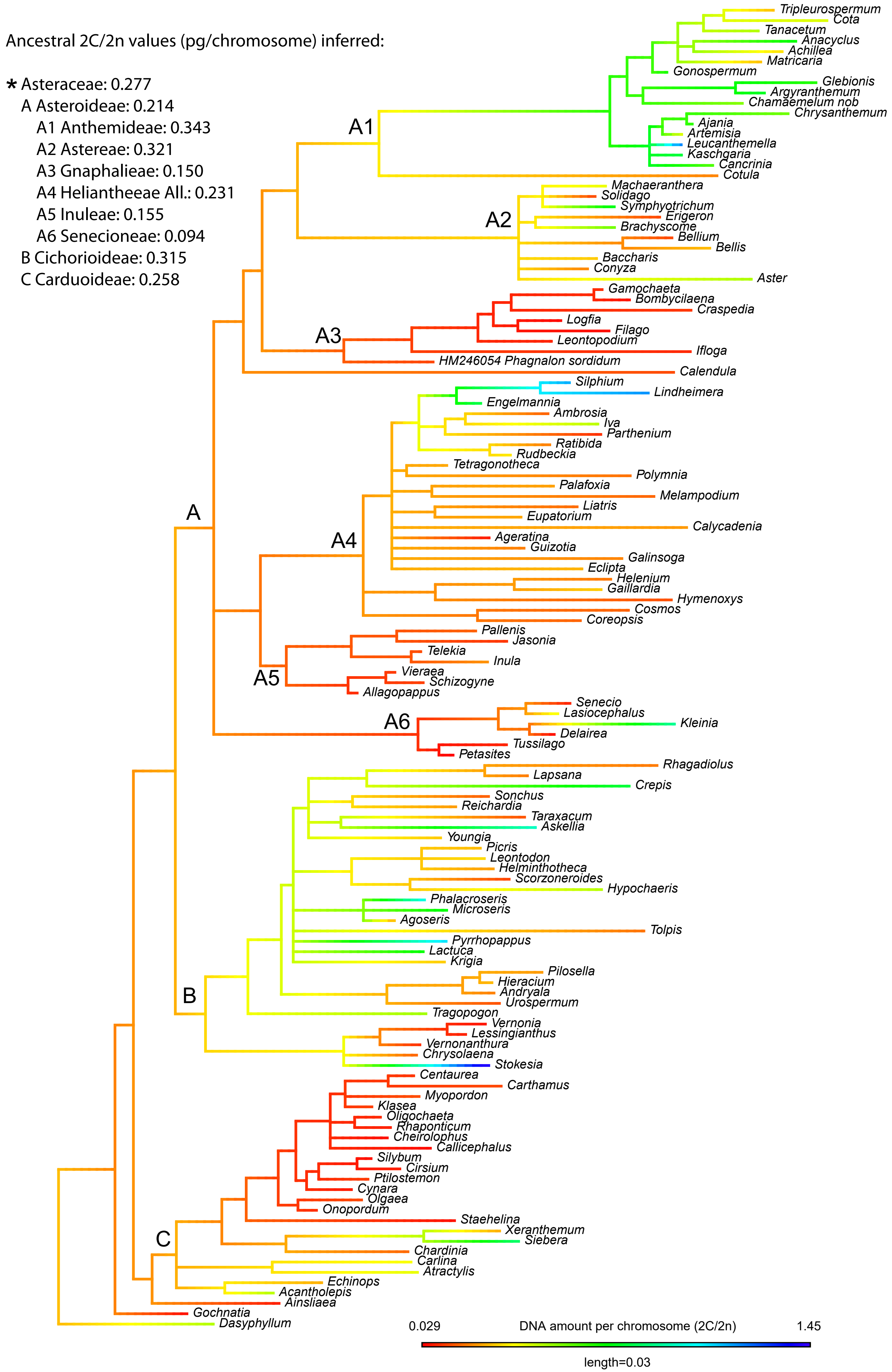
Ancestral 2C/2n values (pg/chromosome) inferred:
* Asteraceae: 0.277
A Asteroideae: 0.214
A1 Anthemideae: 0.343
A2 Astereae: 0.321
A3 Gnaphalieae: 0.150
A4 Heliantheeae All.: 0.231
A5 Inuleae: 0.155
A6 Senecioneae: 0.094
B Cichorioideae: 0.315
C Carduoideae: 0.258
Tripleurospermum
Cota
Tanacetum
Anacyclus
Achillea
Matricaria
Gonospermum
Glebionis
Argyranthemum
Chamaemelum nob
Chrysanthemum
A1
Ajania
Artemisia
Leucanthemella
Kaschgaria
Cancrinia
Cotula
Machaeranthera
Solidago
Symphyotrichum
A2
Erigeron
Brachyscome
Bellium
Bellis
Baccharis
Conyza
Aster
Gamochaeta
Bombycilaena
Craspedia
Logfia
A3
Filago
Leontopodium
Ifloga
HM246054 Phagnalon sordidum
Calendula
Silphium
Lindheimera
Engelmannia
Ambrosia
Iva
Parthenium
Ratibida
Rudbeckia
Tetragonotheca
Polymnia
Palafoxia
Melampodium
A
Liatris
Eupatorium
Calycadenia
A4
Ageratina
Guizotia
Galinsoga
Eclipta
Helenium
Gaillardia
Hymenoxys
Cosmos
Coreopsis
Pallenis
Jasonia
Telekia
Inula
Vieraea
A5
Schizogyne
Allagopappus
Senecio
Lasiocephalus
A6
Kleinia
Delairea
Tussilago
Petasites
Rhagadiolus
Lapsana
Crepis
Sonchus
Reichardia
Taraxacum
Askellia
Youngia
Picris
Leontodon
Helminthotheca
Scorzoneroides
Hypochaeris
Phalacroseris
Microseris
Agoseris
Tolpis
Pyrrhopappus
Lactuca
Krigia
Pilosella
Hieracium
Andryala
B
Urospermum
Tragopogon
Vernonia
Lessingianthus
Vernonanthura
Chrysolaena
Stokesia
Centaurea
Carthamus
Myopordon
Klasea
Oligochaeta
Rhaponticum
Cheirolophus
Callicephalus
Silybum
Cirsium
Ptilostemon
Cynara
Olgaea
Onopordum
Staehelina
C
Xeranthemum
Siebera
Chardinia
Carlina
Atractylis
Echinops
Acantholepis
Ainsliaea
Gochnatia
Dasyphyllum
0.029
DNA amount per chromosome (2C/2n)
1.45
length=0.03
