# Supplementary material for: Progress in the study of genome size evolution in Asteraceae: analysis of the last update
Source: Database (Oxford). 2019 Oct 14;2019:baz098. doi: 10.1093/database/baz098 (PMC6790504; doi:10.1093/database/baz098)
Supplement: Vitales_et_al_Supplementary_Tables_Database_baz098 [file vitales_et_al_supplementary_tables_database_baz098.docx]

**Supplementary Tables**

**Table S1.** A comparison of the number of records for the most represented genera in the GSAD “A Genome Size in Asteraceae Database” (Release 3.0, July 2018). (*) Refers to the increase between the 2^nd^ and the 3^rd^ releases).

|  | **Release 1.0** | **Release 2.0** | **Release 3.0** | **Increment*** |
| --- | --- | --- | --- | --- |
| ***Taraxacum*** | 5 | 90 | 636 | 606,67 |
| ***Artemisia*** | 246 | 366 | 449 | 22,68 |
| ***Hieracium*** | 175 | 260 | 260 | 0,00 |
| ***Crepis*** | 113 | 135 | 204 | 51,11 |
| ***Senecio*** | 185 | 198 | 199 | 0,51 |
| ***Helianthus*** | 97 | 157 | 191 | 21,66 |

**Table S2.** The fit of genome size evolution models in Asteraceae. WN, White Noise, non-phylogenetic model; BM, Brownian motion; OU, Ornstein–Uhlenbeck model; EB, Early Burst model.

|  | **Model** | **-lnL** | **AICc** | **AAICc** | **AICC weight** |
| --- | --- | --- | --- | --- | --- |
| **2C** | **WN** | -397.6856 | 799.463 | 1.2494856 | 0.1924 |
|  | **BM** | -397.060901 | 798.213404 | 0 | 0.3593 |
|  | **OU** | -396.123892 | 798.432399 | 0.2189952 | 0.3221 |
|  | **EB** | -397.060901 | 800.306417 | 2.0930125 | 0.1261 |
| **2C/2n** | **WN** | -24.091421 | 52.278842 | 22.8294358 | 0.054912 |
|  | **BM** | -12.676703 | 29.449406 | 0 | 0.49776 |
|  | **OU** | -12.045529 | 30.284607 | 0.8352004 | 0.3278 |
|  | **EB** | -12.67670 | 31.546955 | 2.0975481 | 0.174398 |

**Table S3.** Results of phylogenetic signal estimators for genome size values (2C) and DNA amount per chromosome (2C/2n) on diploid Asteraceae dataset.

|  | **Genome size (2C)** | | **DNA amount per chromosome (2C/2n)** | |
| --- | --- | --- | --- | --- |
|  | **value** | **p-value** | **value** | **p-value** |
| **Pagel’s λ** | 0.6884 | 1.4971e-06 | 0.8839 | 4.9926e-08 |
| **Bloomberg’s K** | 0.3374 | 0.044 | 0.4107 | 0.041 |
| **C mean** | 0.3021 | 0.001 | 0.3066 | 0.001 |
| **Moran’s I** | 0.05656 | 0.001 | 0.05602 | 0.001 |
